# Association Between Appendectomy and *Clostridium difficile* Infection

**DOI:** 10.4021/jocmr770w

**Published:** 2012-01-17

**Authors:** Rashida Merchant, William R. Mower, Ariel Ourian, Fredrick M. Abrahamian, Gregory J. Moran, Anusha Krishnadasan, David A. Talan

**Affiliations:** a The UCLA Emergency Medicine Center, UCLA Geffen School of Medicine, Los Angeles, CA, USA; b Department of Emergency Medicine, Olive View-UCLA Medical Center, Sylmar, CA, UCLA Geffen School of Medicine, Los Angeles, CA, USA

## Abstract

**Background:**

Recent theory proposes that the appendix functions as a reservoir for commensal bacteria, and serves to re-inoculate the colon with normal flora in the event of pathogen exposure or purging of intestinal flora. If true, we reasoned that flora from a normal appendix could provide protection against *Clostridium difficile*. We conducted this investigation to examine the protective effect of an intact appendix and test the hypothesis that prior appendectomy will be more common among patients with a positive test for *C. difficile* as compared with patients who test negative.

**Methods:**

We contacted patients who had undergone *C. difficile* testing and asked them whether or not they had a prior appendectomy. Using their responses and results from Toxin A & B EIA tests, we calculated the difference in appendectomy rates between those who tested positive for *C. difficile*, and those who tested negative. We considered a positive 15% absolute difference to represent a significant increase in appendectomy rate.

**Results:**

We enrolled 257 patients. Among the 136 who tested positive for *C. difficile*, 27 (19.9%) had prior appendectomies, while among 121 patients testing negative for *C. difficile*, 38 (31.4%) had prior appendectomies, yielding a difference in appendectomy rates of -11.6% (95% Confidence Interval: -21.6% to -0.9%).

**Conclusions:**

The rate of prior appendectomy was actually lower among patients with a positive *C. difficile* test as compared to those with a negative test. Conversely, patients who tested positive for *C. difficile* were more likely to have an intact appendix than those who tested negative. These results suggest that rather than being protective, an intact appendix appears to promote *C. difficile* acquisition, carriage, and disease.

**Keywords:**

*Clostridium difficile*; Appendix; Appendectomy; Microbial reservoir; Infection

## Introduction

The vermiform appendix is theorized to serve as a microbial reservoir that facilitates re-inoculation of colonic bacteria after serious gut infections [1,2]. The appendix can harbor both commensal and potentially pathogenic bacteria and allow these organisms to live in a state of biofilm, with the various microbes existing in a symbiotic relationship [1]. Additionally, the location of the appendix may contribute to its function as a reservoir for normal colonic bacteria since it provides somewhat of a distance and barrier from feces and contamination from potential pathogenic organisms [1,2]. The idea that the appendix has the ideal environment to house enteric bacteria in biofilms is plausible, and its role in immune protection may be based upon providing normal colonic flora in times of need [1,2].

The bacterium C. difficile is the most common cause of nosocomial diarrhea, with rates of *C. difficile* associated disease (CDAD) increasing, particularly among the elderly, adults, and children [3-7]. The generally accepted theory for the pathogenesis of *C. difficile* involves the disruption of normal intestinal flora, typically in the setting of prior antibiotic use, which allows *C. difficile* to proliferate [5].

If the concept that the appendix serves a protective function as reservoir for normal colonic bacteria is true, then it seems plausible that the risk of infections with pathogenic bacteria, such as *Clostridium difficile*, will increase among patients who have undergone prior appendectomy as compared with patients who have an intact appendix. We therefore hypothesized that the rate of prior appendectomy would be higher among patients who test positive for *C. difficile* as compared with the appendectomy rate among those testing negative.

## Materials and Methods

We expected the prevalence of *C. difficile* infection to be relatively low among our tested population and therefore elected to employ case-control methodology to expedite our work and preserve efficiency. We also wanted to examine appendectomy rates among a ”normal“ population. These considerations required us to identify three separate populations; our case series, our controls, and our ”normals.“

We identified our case and control populations by reviewing pathology results from our institution and identifying all patients over 18 years of age who underwent clinical testing for *C. difficile* using Toxin A & B EIA tests between July 2006 and December 2008.

We classified patients as positive for *C. difficile* infection if test results on their initial specimen were positive. These patients formed our case series. We considered patients to be free of *C. difficile* infection (negative for disease) if their specimens tested negative. We constructed our control population from among patients with negative results by selecting individuals matching the age (within two years), sex of each patient in our case series.

We constructed our ”normal“ population from patients who presented to our institutional ambulatory care center with non-gastrointestinal complaints and who did not undergo *C. difficile* testing. From among this group we enrolled two individuals matching the age and sex of each patient in our case and control series.

We surveyed all participants and asked them whether or not they had undergone an appendectomy. We used these responses to calculate the proportion of patients who had undergone prior appendectomy in each of our three populations. To test our primary hypothesis, we calculated the confidence interval for the difference in prior appendectomy rates between the case (patients testing positive for *C. difficile*) and control (patients testing negative for *C. difficile*) populations. We considered our hypothesis to be confirmed if the lower confidence interval for this difference was positive.

We designed our study to have an 80% power to detect a 15% absolute increased prevalence of in appendectomy rates under one-sided testing of a 95% confidence interval. We used statpages.org for our statistical calculations [8]. The study was reviewed and approved by our institutional review board.

## Results

We enrolled 853 patients in our study, including 17 patients who could not recall whether they had undergone prior appendectomy. After excluding these individuals from further analysis, our final population of 836 patients included 136 patients who tested positive for *C. difficile* (case population), 121 patients testing negative (control population), and a ”normal“ population of 569 patients.

Patients in our case series ranged in age from 20 to 99 years (median 59; interquartile range: 49 to 72), and included 68 males (50.0%) and 68 females (50.0%). Our control group ranged from 18 to 96 (median 61; interquartile range: 47 to 72) with 57 males (47.1%) and 64 females (52.9%). Our ”normal“ population ranged in age from 18 to 102 years (median 62; interquartile range: 48 to 76) with 265 males (46.6%) and 304 females (53.4%).

Among the 136 cases (patients who tested positive for *C. difficile*), 27 had prior appendectomies (19.9%), while among the 121 controls (patients who tested negative for *C. difficile*), 38 had a prior appendectomy (31.4%), yielding a difference in appendectomy rates of -11.6% (95% C.I.: -21.6% to -0.9%). In our ”normal“ population, 105 patients had prior appendectomies (18.5%).

## Discussion

Our results show that the rate of prior appendectomy was actually lower among patients who tested positive for *C. difficile* as compared with patients who tested negative. This result contradicts our primary hypothesis, and indicates that patients who tested positive for *C. difficile* were more likely to have an intact appendix than those who tested negative.

These results probably reflect some naivete on our part regarding the true function of the appendix. While our study cannot show causality, a possible interpretation of our findings would be that the appendix is indeed a microbial reservoir, and that *C. difficile* is among the harbored organisms. Under the selective pressure of antimicrobials, *C. difficile* is able to emerge and colonize the lower gut. Conversely, the higher rates of prior appendectomy we observed among patients with negative *C. difficile* tests may be due to the lack of an appendix to harbor *C. difficile* and promote its inoculation into the gut. Thus it appears that rather than providing protection against CDAD, an intact appendix may promote *C. difficile* acquisition, carriage, and disease.

Our study provides additional support for the concept of an appendiceal microbial reservoir. We observed the highest rate of appendectomy, 31.4%, among our control population (patients who had negative test results). The rate of appendectomy among this population exceeded the rates for our case population, as well as the 18.5% appendectomy rate among our ”normal“ population. This high rate of appendectomy among patients with non-CDAD may reflect difficulties in re-colonizing the lower gut and is consistent with the idea that the appendix plays an important role in re-establishing normal bacterial colonization.

It is also worth noting that the only a small minority of colitis patients develop *C. difficile* infections. Thus the benefits of gut re-colonization and prevention of colitis occur much more frequently than pathogenic *C. difficile* infections. This is likely to be important from an evolutionary perspective where the substantial infirmity that arises from appendicitis is balanced by benefits from an intact appendix and overall increased survival [2]. With this in mind, it may be possible to implement treatments for acute appendicitis, such as selective antibiotic therapy, that reduce the morbidity and mortality of appendicitis, while preserving the appendiceal microbial reservoir [9,10].

### Limitations

Our choice of case-control methodology makes it impossible for us to demonstrate causality. Our observed associations may not be due the presence or absence of an appendix, but may reflect biological processes that alter both the risk of developing appendicitis, and risk of developing CDAD. Demonstrating causality will be difficult, as a definitive randomized controlled trial of appendectomy is unlikely to be conducted in the foreseeable future.

Our methodology also makes it impossible for us to determine the prevalence of *C. difficile* among our populations. This information may improve our understanding of the relationship between *C. difficile* and appendectomy, but was not essential to the primary purpose of this investigation. We are also limited in our ability to examine how infection and appendectomy rates vary as a consequence of important variables such as prior antibiotics use, history of prior bowel dysfunction, age, duration of symptoms, and others.

Our results also likely overestimate appendectomy rates among our case population. This is a reflection of the imperfect specificity of the toxin A & B EIA test and the potential for the false positive rate to attain a magnitude similar to the prevalence of CDAD. The false positive cases are likely to have appendectomy rates similar to the true negatives (31.4% in this case), and increase the observed appendectomy rates among our case population. Eliminating this source of bias would likely lead to an even stronger association between the CDAD and the presence of an intact appendix.

### Conclusions

The rate of prior appendectomy was lower among our patients who test positive by EIA for *C. difficile* toxin as compared with patients who test negative. At our institution, more than 80% of *C. difficile* infections occurred in patients who had an intact appendix.
